# Spray Pyrolysis of Ternary Oxides: From Precursor Selection to Surface Reactions

**DOI:** 10.3390/ma19153305

**Published:** 2026-08-04

**Authors:** Karsten Fleischer, Priyanka Bhatnagar, Ciarán Cooling, Eva Gurley, Dominik Jakobczak, Ainur Zhussupbekova

**Affiliations:** 1 School of Physical Sciences, Dublin City University, D09 V209 Dublin, Ireland; 2 School of Physics and Centre for Research on Adaptive Nanostructures and Nanodevices (CRANN), Trinity College Dublin, D02 PN40 Dublin, Ireland; 3 L.N. Gumilyov Eurasian National University, Astana 010000, Kazakhstan

**Keywords:** spray pyrolysis, transparent conducting oxides, thermogravimetric analysis, solubility, thin-film homogeneity, p-type TCO, amorphous TCO

## Abstract

Spray pyrolysis is a popular method for the low-cost synthesis of oxides and other compounds in thin-film form. Here, we discuss several nuances of using this method for ternary and quaternary compounds. Specifically, we outline how precursor solubility and thermal decomposition, as well as desorption of intermediate species from the sample surface govern the spray pyrolysis growth process. We demonstrate how the stoichiometry transfer from solution to film can be affected in selected test cases. We present real-time optical growth measurements of the behaviour of individual Cu, Cr, Zn, and Sn precursors, as well as post-growth analysis of film composition by X-ray photoelectron spectroscopy for ternary transparent conducting oxides (TCOs; p-type: Cu_x_CrO_2_, SnTiO_*x*_; and n-type: a-ZnSnO_3_). We illustrate how several steps of spray pyrolysis affect the stoichiometry transfer from the solution to the ternary thin film. Using binary Cu_2_O as a test case, we also show how the choice of instrument geometry and nozzle type can affect film homogeneity. All materials discussed have been chosen to highlight potential difficulties of the spray pyrolysis process of ternary, quaternary, or even more complex oxides, and the mechanisms should be considered for other materials as well. We therefore also provide an extensive overview of suitable precursor salts with similar expected properties as used in this experimental work to guide future ternary oxide studies.

## 1. Introduction

The replacement of critical rare raw materials in modern technology with stable, cost-efficient, environmentally safe, earth-abundant alternatives is one of material sciences central challenges. The dependence of technologies, such as computer memory/storage, energy efficient lighting, light emitting diodes (LEDs), high resolution thin-film transistor (TFT) displays, organic LED (OLED)-based displays on scarce materials, raises concerns about security of supply, costs to consumers, sustainability, and, in some cases, toxicity. There has been considerable effort in recent years to develop material screening processes—experimental and computational—to find new, well-performing materials from abundant constituent elements only.

In this context, experimental screening of novel ternary oxides using low-cost deposition techniques is a popular research field. While the properties of most basic binary oxides are well known, there is growing interest in screening the properties of ternary and quaternary materials of various crystal structures, for instance, spinel, delafossite, and perovskite oxides. One popular method to rapidly alter the composition of a thin film is spray pyrolysis (SP) [1,2,3]. In SP, metal salts are dissolved in a solvent such as water or ethanol. Since the solution stoichiometry can be easily changed, so too can the stoichiometry of the grown thin film, making the technique suitable for rapid screening. The initial solution is nebulised using a liquid flow and compressed gases in various types of nebulisers [1,2,3]. The solvent/salt/gas mixture then thermally decomposes and reacts near a heated substrate surface to form a thin film. Depending on the choice of precursor and carrier/nebulising gas, metals, oxides, sulphides, and other components can be synthesised at very low costs as the method is carried out at ambient pressure with no need for expensive vacuum infrastructure. The method itself is well known, and used extensively in research labs and in some selected industrial applications, specifically for the synthesis of oxides for large-area applications.

In this article, we will discuss specific challenges of the SP technique when investigating more complex ternary or quaternary oxides:

- The choice of precursor when synthesising ternary materials in general.
- The choice of precursor when aiming to reduce synthesis temperature.
- Temperature- and precursor-chemistry-induced effects leading to non-stoichiometric transfer of solution to thin film cation ratios.
- The influence of spray geometry and solvent choice on film composition and homogeneity.

The main aim of this article is not to give a comprehensive growth optimisation or thin-film characterisation of the specific materials but to use examples from our work on transparent conducting oxides to highlight the possibilities of often under-reported issues specific to the spray pyrolysis process itself. We discuss how differences in the spray geometry and precursor specific mechanisms can lead to non-stoichiometric transfer of the solution cation ratio into the spray pyrolysis-grown ternary oxide film.

Issues will be demonstrated exemplarily for the synthesis of ternary transparent conducting oxides (TCOs). However, many aspects of the additional challenges of the ternary materials synthesis discussed in this work can be equally important for other materials and applications. The effects discussed here should be considered when choosing spray pyrolysis for ternary oxide synthesis.

One of the major advantages of spray pyrolysis is that it is done in atmospheric conditions, significantly reducing instrumentation costs. The downside of this is that commonly applied in situ characterisation methods employed in physical vapour deposition (PVD) and chemical vapour deposition (CVD) systems are simply not available. Even optical methods are rarely employed due to the need to encase the growth system and the light scattering caused by the mist of the precursor solution, which disturbs in situ optical methods. Some groups have employed reflectometry, but only in thick films, providing information on the thickness of the grown film, not on the growth mechanism or surface reactions themselves [4]. For this reason, our group developed a method to employ refractive index sensing Au-dimers to obtain some insight into the initial growth process and precursor adsorption onto the substrate surface [5]. While a powerful method, the concept is based on changes in the refractive index in the vicinity of Au-dimers and hence, is not species-selective. Therefore, it does not allow us to directly infer the stoichiometry if ternary materials are grown. As a result, we only use it here to investigate the onset of the spray pyrolysis process on the substrate surface for selected Cu, Cr, Zn, and Sn precursors.

Typically, the synthesis of ternary compounds is analysed by post-growth methods, for instance, X-ray diffraction (XRD), where the compound stoichiometry is confirmed by pattern matching. There are many examples where spray grown ternary materials show good crystalline quality and a transfer of the solution cation ratio into the thin-film stoichiometry. Examples include zinc-blende, perovskite, spinel, and other ternary oxides [1,6,7,8,9,10,11,12,13], even mixed chalcogenides [14]. XRD infers stoichiometry by comparing measured d-spacings with reference samples. However, the peak positions can be influenced not only by altered stoichiometry but also strain and reduced grain sizes. Partial de-phasing of materials, specifically into amorphous phases at low growth temperatures, also cannot be quantified by XRD. Most reports focus on a working combination of precursors and growth conditions without reporting on specific differences or unsuccessful synthesis routes. Hence, effects discussed in this work are often under-reported.

We deliberately work to lower growth temperatures to reduce the thermal budget of device manufacturing and therefore often analyse ultra-thin films (<50 nm), nano-crystalline materials, and even amorphous oxides. For these types of samples, XRD is often not helpful in assessing the film stoichiometry. Instead we use X-ray photoelectron spectroscopy (XPS) to directly determine the thin-film stoichiometry of the ternary compounds (see Supplementary Information (SI)). XPS is sometimes used to analyse SP-grown films and there are examples where stoichiometric transfer from the solution was confirmed [7,8,13], but also cases where smaller variations in the thin-film stoichiometry are observed [14]. In some cases, very large variations can occur, often highly dependent on growth conditions [15,16,17]. In this paper, we will demonstrate the latter and provide examples of several mechanisms leading to such a non-stoichiometric transfer.

## 2. Influence of Precursor Selection

**Solubility:** The first step in any thin-film synthesis by spray pyrolysis is dissolving metal salts in the solvent of choice. Ideally, the film growth rate will be proportional to the molarity of the metal cation in the solution. For binary materials, changing the molarity typically only alters the growth rate and indirectly crystallinity, as well as the roughness of the film [3,18,19,20,21,22,23]. In general, reducing the molarity, while decreasing growth rate reduces film roughness, increases crystalline quality and size, and can induce crystallographic texture as crystal planes with the highest growth rate can dominate.

For ternary compounds, additional considerations have to be taken into account. The most straightforward consideration is the selection of precursors in terms of their solubility. When using salts of a similar type (i.e., only chlorides: *A*Cl and *B*Cl) there are typically no additional complications other than ensuring that the total molarity does not exceed the solubility limit of one of the salts.

Additional issues may arise when different types of salts are utilised. When using two types *AX* and *BY*, it is essential that all combinations *AX*, *AY*, *BX*, *BY* are sufficiently soluble as otherwise fallout of one component can occur when both salts are put into the solvent. Table 1 gives an overview of some precursor salts with low decomposition temperature and suitable solubility in water, ethanol, or methanol to be of use in spray pyrolysis. For water-based solutions, the maximum molarity is given and for ternary precursor mixtures the total molarity of the sum of the cation species needs to be below the maximum solubility of the least-soluble compound.

Spray pyrolysis recipes can utilise additional chemicals to increase solubility of the precursor. Adjusting the pH of the solution is, for instance, a popular method. However, as this is highly specific to each precursor salt, it is beyond the scope of this work.

In general, solutions close to the solubility limit should be avoided in spray pyrolysis, as it can lead to additional complications when using spray blast nozzles. As the solution is nebulised by an expanding gas, the solution is cooled down at the nozzle, reducing solubility and causing fallout and build-up of unreacted precursors at the nozzle. This can dramatically change the droplet sizes leading to inconsistent growth across different runs. More importantly for ternary and quaternary compounds, it can lead to significant changes in the relative concentration of the different cations.

**Precursor Decomposition:** The other important property of a spray pyrolysis precursor is the temperature at which thermal decomposition begins. Typically SP systems operate between 150 °C and 500 °C and it is important that the precursor readily decomposes at these temperatures. For growth of binary oxides, the situation remains relatively simple: the decomposition temperature of the precursor determines the minimum temperature upon which growth occurs. Increasing the temperature leads to increased growth rates and improved crystal quality. At excessively high temperatures, growth can be limited due to reactions happening too far away from the substrate surface, or increased desorption of suboxide species from the thin-film surface (see below).

When growing ternary compounds, however, the incorporation rate of different cations now depends on the decomposition rate for two different precursor species. For a given temperature, in the extreme case, one precursor may be fully decomposed, while another is not at all. In such cases, the thin-film stoichiometry will substantially differ from the relative molarity of the species in solution. As a general rule, one needs to select precursor salts with compatible solubilities and similar decomposition temperatures for a predictable transfer of solution molarity into thin-film stoichiometry.

**Figure 1:**
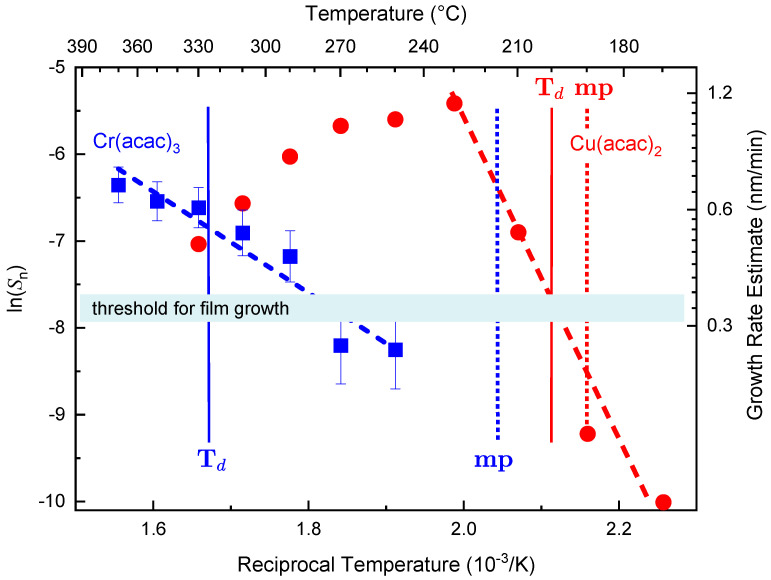
Spray pyrolysis growth rate of Cu_2_O and Cr_2_O_3_ as a function of temperature using metal acetylacetonates in methanol. The threshold temperature for SP growth follows expectations from the thermal decomposition data (see Table 1) and is seen between the melting point (**mp**) and onset of thermal decomposition (**T_*d*_**). For the copper precursor, a plateau in growth rate is observed once the precursor fully decomposes. For the chromium precursor, the temperatures achievable in the system were not high enough to observe this.

The following example will demonstrate this issue for the case of copper-deficient delafossite Cu_x_CrO_2_ (x∼0.4), an interesting material for applications as a p-type transparent conducting oxide [17,59,60]. Best-performing spray pyrolysis-grown Cu_x_CrO_2_ was grown using metal acetylacetonates in methanol (Cu(O_2_C_5_H_7_)_2_, Cr(C_5_H_7_O_2_)_3_) [17]. Both precursors are only slightly soluble in methanol and the overall molarity in the solution was hence kept below 0.015 M to avoid fallout of precursors in solution. Despite this, it was seen that the thin-film composition can be significantly different from the solution molarity, partially due to the substantial difference in decomposition temperatures for the two precursors. Figure 1 shows the results of an *in situ* study of binary oxide formation in spray pyrolysis, confirming the close relationship of the precursor decomposition temperature with the onset of film formation in spray pyrolysis [5].

Using the measurements for the precursor decomposition and binary oxide formation, one can already see that a stoichiometric transfer from the solution to a ternary film can only occur when the growth temperature is in a window where growth rates for the two respective binary components are comparable.

**Figure 2:**
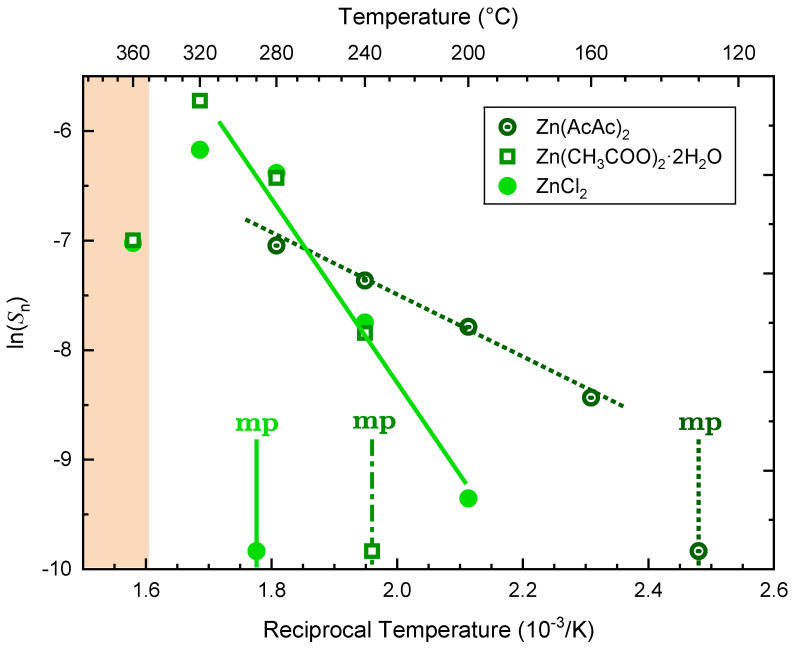
Spray pyrolysis growth rate of ZnO as function of temperature using various Zn salts in methanol. For the acetylacetonate, the melting point and thermal decomposition (see Table 1) are good indicators of the onset of growth. For the acetate and chloride, the growth rate is very similar despite significant differences in thermal decomposition temperatures.

Figure 2 shows a similar study for various Zn salts, using the same method to evaluate the effectiveness of such salts as spray pyrolysis precursors.

The behaviour of zinc acetylacetonate is very similar to that observed for the Cu and Cr equivalents. The onset of ZnO growth is closely correlated with the melting point and the thermal decomposition of the actual salt. However, for alternative salts, we see variations of this trend. The rates for zinc chloride or zinc acetate are very similar, and growth occurs even well below the thermal decomposition temperature of ZnCl_2_, which is above 450 °C. Although a reduction in growth rate was observed above 350 °C, this is attributed to the instability of the gold nanoparticles employed for growth sensing rather than a substantial growth-rate reduction. Zinc acetate is frequently employed for ZnO film growth even at much higher growth temperatures around 450 °C [61,62,63]. We attribute the onset of growth well below the thermal decomposition of ZnCl_2_ to a substantially different growth mechanism. For acetylacetonates, the salt decomposes on the growing surface after a physisorption process, while ZnCl_2_ is fully dissociated and Zn^2+^ ions are oxidised in the gas phase leading to the adsorption of ZnO onto the surface. We expect the same for the acetate and hence the same overall growth dynamic is observed.

**Figure 3:**
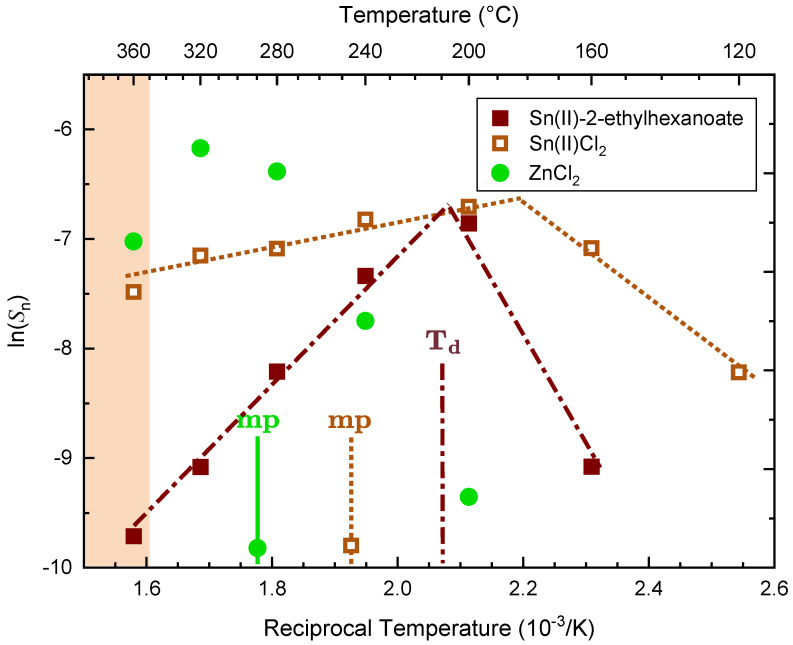
Spray pyrolysis growth rate of SnO_2_ as a function of temperature for two common Sn salts. The ZnO growth values for ZnCl_2_ are also given as reference. The different behaviour of these precursors directly explains why changing precursors is expected to alter the thin-film stoichiometry even dramatically if solution molarities are kept constant.

All these zinc precursors have been previously employed in conjunction with tin precursors to grow ternary, amorphous Zinc Tin Oxide (a-ZTO) [16]. A large variation of film properties was observed when nominally similar Zn/Sn ratios have been used in the solution. If one compares the measurements of binary SnO_2_ and ZnO growth in Figure 3, it is evident that incorporation rates are expected to differ significantly for different precursor salts. For the growth of a ternary compound, this implies that the ratio of Zn/Sn in solution will not be stoichiometrically transferred into the film. With electrical properties depending strongly on Zn/Sn ratio within the film, this behaviour then directly explains the large variations in film properties when changing precursors.

For lower temperatures, the dominant effect observed in our *in situ* studies is the thermal decomposition of the original precursor. While we only investigated selected Cu, Cr, Zn, and Sn precursors, the precursor decomposition is typically known from thermogravimetric analysis (TGA). Indeed, for the investigated precursors, the observed behaviour follows the expectations from TGA. In Table 1, we have therefore included the measured TGA decomposition temperatures if they are known for the precursor salt.

In general, our measurements show that the increase in growth rate with temperature will reach a saturation point when all molecules are decomposed. If the temperature is further increased, one would expect a plateau of constant growth rate. However, only for some cases like the Cu(AcAc)_2_ or SnCl_2_ is such a plateau is observed. For most of the investigated precursors, a decline in growth rate is measured when temperatures are further increased. This is caused by a combination of two different processes: depletion and desorption.

**Depletion:** Depending on the reactor geometry the decomposition process can occur already further away from the sample surface. Such reactions can lead to the formation of nanoparticles within the gas, which, depending on reactor geometry can either become incorporated into the growing layer or leave through the chamber exhaust. These reactions within the gas phase effectively reduce the amount of precursors available at the sample surface and reduce the growth rate.

**Figure 4:**
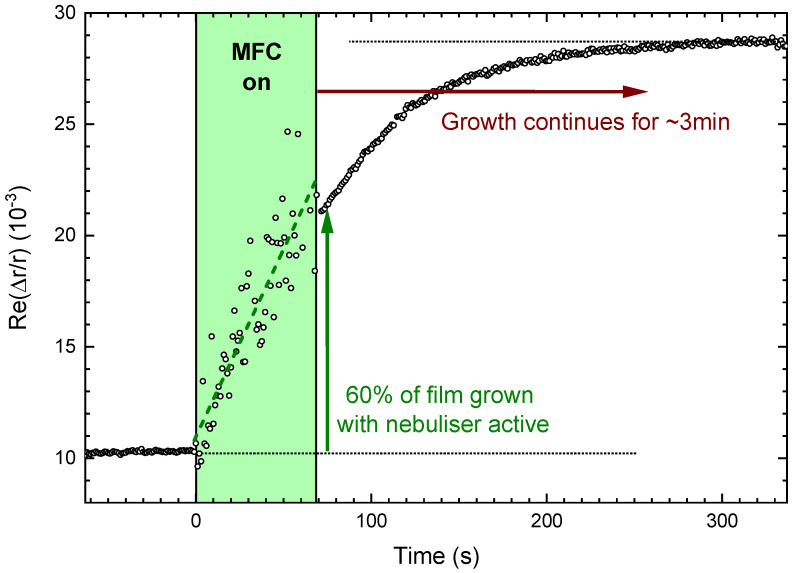
RAS signal taken during deposition of ZnO using ZnCl_2_ as precursor. When the nebuliser is operated (MFC gas flow enabled for 70 s), a linear change is observed, proportional to the film deposition. The noise is significantly increased due to the “waves” of mist from the nebuliser crossing the optical path. Once the gas flow is stopped the vaporised solution remains in the chamber and the film continues to grow.

Figure 4 shows a transient measurement of the reflectance anisotropy signal monitored for the *in situ* measurements. When the nebuliser is activated, the occurring growth leads to a linear change in signal. When the nebuliser is turned off, we see a continuation of the growth for an extended time, with very little rate change for the initial 10–20 s after switch off. Hence, growth continues even after the supply is cut, meaning the precursor is available within the gas phase for an extended time after the gas purging slowly removes it from the chamber. In the case of a depletion-limited growth, cutting off the supply would lead to an immediate growth stop. We have to stress that while gas-phase depletion was not an issue in our reactor design, in other designs, particularly for large chambers with larger nozzle to sample separation and larger heaters, this mechanism needs to be considered. In case bilayers are grown or to prevent surface composition gradients, one can evacuate the growth chamber to remove the precursors and end the growth more sharply.

**Desorption:** Another important aspect of the spray pyrolysis growth process is the desorption of precursors, suboxides, and the material grown. The details of these processes differ significantly between precursors and grown materials, and are highly temperature-dependent. In all chemical vapour phase deposition (CVD) methods, reactant desorption is of importance. In spray pyrolysis, despite being a CVD-like growth process, there are very few detailed studies of desorption processes available and we can only give some generic arguments here.

In Figure 1, it is very noticeable that the copper oxide growth rate reduces at higher temperatures. This behaviour is commonly observed in other growth methods like Molecular Beam Epitaxy or Chemical Vapour Phase Deposition [64,65] and is therefore likely a signature of the same process—the removal of volatile suboxides or the grown material itself from the film surface. Hence, less precursor material is incorporated at higher temperatures.

This behaviour is seen for all the precursors investigated here. Only in the case of chromium acetylacetonate is this desorption not observed, though this is only due to the limited temperature window in which we are able to perform these measurements. At higher temperatures, one would expect the same behaviour.

In binary systems, this desorption process only influences the growth rate. For ternary or more complex oxides, the desorption process can differ between suboxides of the individual cations and this process may therefore lead to significant differences in the transfer of the solution stoichiometry into the film stoichiometry.

## 3. Surface Reactions

**Figure 5:**
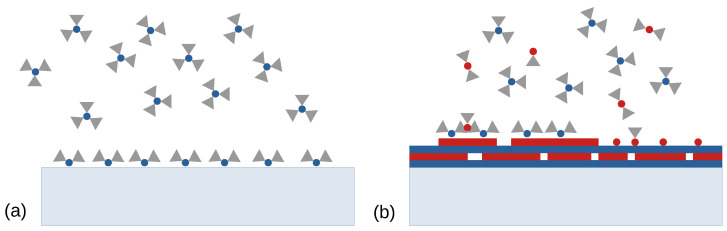
Schematic illustration of the CuCrO_2_ growth process. (**a**) illustrates the situation at 310 °C if solely Cr(acac)_3_ (blue marked molecules) is provided: no growth occurs, the precursor only partially decomposes and there is self-limited adsorption. (**b**) shows how the situation changes if both Cr(acac)_3_ and Cu(acac)_2_ (red marked molecules) are provided: Cr(acac)_3_ readily reacts with surface CuO_x_, the Cu(acac)_2_, or fully decomposed CuO_x_ reacts with the surface-adsorbed Cr(acac)_3_ leading to a continued, layered oxide growth. (solid red/blue regions).

To date, we have discussed how the behaviour of individual precursors differs. To complicate things further there are also different mechanisms by which cross-reactions at the film surface between the different cations and anions of the precursor as well as their suboxides or partially decomposed precursors can alter the growth of ternary oxides. The details depend strongly on the individual oxide and precursors and we can therefore only discuss selected examples of such interactions in three ternary oxide examples:

**CuCrO_2_:** In the case of CuCrO_2_, or to be precise, copper-deficient Cu_x_CrO_2_, precursor and suboxide interactions at the growing surface play a crucial role, and the growth of the ternary compound significantly differs from expectations derived from studies of binary oxides. The ideal growth temperature to grow the p-type transparent oxide Cu_x_CrO_2_ in spray pyrolysis is found to be in the narrow range of 310–325 °C, depending on the geometry of the growth chamber. Based on the studies of the individual precursors from Figure 1, this is surprising, as this is well below the temperature where Cr_2_O_3_ could be synthesised using the Cr(acac)_3_ precursor, while at the same time it is well above the optimum temperature of growing Cu_2_O using Cu(acac)_2_. Figure 5b illustrates how it is exactly the interaction of the precursor and surface suboxides that leads to the formation of crystallographically oriented Cu_x_CrO_2_, as the film is built layer by layer continuously, while the adsorption of Cr(acac)_3_ is self-limiting at this temperature [66], the same precursor readily binds to copper suboxides at the film surface. At the same time, the copper suboxides, which readily leave the surface, are prevented from doing so by the coverage of the chromium precursor. Thus, a film is continuously formed at growth rates significantly larger than expected for either binary oxide. In this example, the on-surface reactions *enable* the film formation.

**SnTiO_x_:** In the SnTiO_3_ stoichiometry, the material is predicted to be a high mobility p-type TCO and we present early attempts to synthesise the material. The exact phase of the films has not yet been confirmed, but we do observe large variations in stoichiometry depending on the choice of precursors. While in the previous example, the presence of the Cu suboxide on the film surface promoted Cr incorporation (and vice versa), we see an entirely different behaviour when growing SnTiO_x_ using TiCl_3_ and SnCl_2_ as precursors. For such simple inorganic salts, the precursor salt dissociates fully within the solution. The oxide growth process in the gas phase and on the surface is facilitated by oxidation reactions of the cations involving residual oxygen from the growth atmosphere or -OH groups from the solvent. For the specific precursor combination, both chlorides are highly soluble in methanol, and as the anion (Cl) is the same, no unwanted cross-reactions within the solution occur.

**Figure 6:**
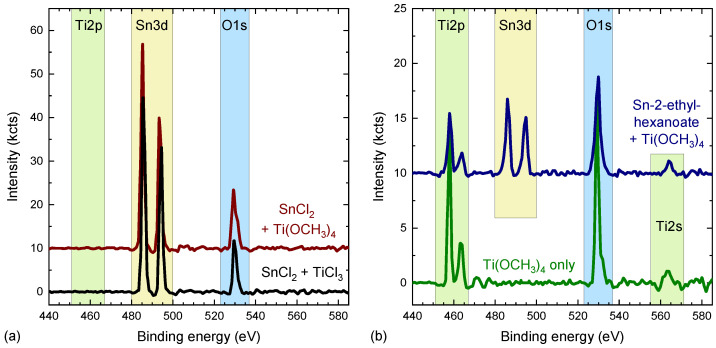
XPS analysis of targeted samples fabricated using a combination of inorganic and organic Ti and Sn precursors. (**a**) If SnCl_2_ is used as the tin source, no titanium is incorporated independently of the choice of Ti precursor (**b**) Titanium (IV) methoxide and SnCl_2_, highlighting the role of Cl-based precursors in suppressing titanium in the growing films.

However, no Ti was observed in XPS measurements of the resulting films, whereas clear tin incorporation was observed (Figure 6a). To verify the viability of TiCl_3_ as a valid precursor, a film was deposited using only TiCl_3_—again no Ti signal was detected, suggesting the precursor itself is unsuitable. When replaced by Titanium (IV) methoxide, we clearly observe the formation of TiO_2_ when used as sole precursor at 380 °C (Figure 6b). However, when mixed with SnCl_2_, the Ti signals disappeared again, and only tin oxide grows. To confirm whether Ti was buried below the possible Sn-rich surface, Argon etching treatment (3 min total in 30 s steps) was carried out, but no Ti was seen even in the case of the 5:95 ratio of Sn:Ti. This strongly confirms that Ti incorporation into the film is completely suppressed in the presence of Cl-ions in the solution. This disappearance of Ti in all chlorine-based precursors indicates that the presence of chlorine strongly influences the film growth chemistry during spray pyrolysis. We also examined the Cl 2p region for the samples prepared by using chloride-based precursors, which showed weak or no detectable Cl signal. A weak Cl 2p signal was observed for the films prepared from solutions containing TiCl_3_, whereas no detectable Cl 2p signal was observed for films with SnCl_2_-Titanium (IV) methoxide combination (see Supplementary Materials for further details). This suggests that chloride primarily influences the precursor decomposition and film growth chemistry rather than being incorporated into the final film. Only by changing to organic precursor salts for both Sn and Ti (Sn(II) 2-ethylhexanoate and Titanium (IV) methoxide) can this situation can be changed for this particular ternary oxide. The XPS of the film deposited by organic precursors is shown in Figure 6b, which shows equal incorporation of Sn and Ti, close to the solution stoichiometric ratio.

The possible explanation for the disappearance of Ti in all Cl-based precursors could be the chlorine rich environment formed under spray conditions. There are reports showing that TiCl_3_ disproportionates to form volatile TiCl_4_ [67]. Since the boiling point of TiCl_4_ is 136 °C [53], which is lower than the current deposition temperature (380 °C), there is a chance that Ti is lost through gas-phase reactions before it reaches the substrate. A similar reaction might be occurring when TiCl_3_ is mixed with SnCl_2_, where SnCl_2_ would be providing additional chloride that facilitates the formation of volatile TiCl_4_. Although TiCl_4_ formation was not directly observed in the present work, this hypothesis is consistent with the complete absence of Ti in XPS for all chloride-based precursors even at high Ti:Sn ratios, whereas Ti is incorporated normally in chloride-free precursors. This specific example highlights that a soluble precursor, with a low decomposition temperature, does not guarantee that it can be used for spray pyrolysis. Certain combinations of cations with the anions of a second precursor can also fully suppress incorporation.

**Figure 7:**
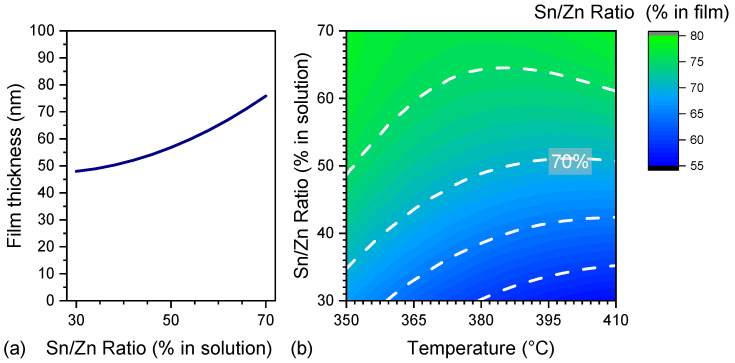
Examples of the non-linear transfer of the Sn/Zn ratio within the solution into the thin films. (**a**) shows the main effect of greater overall film thickness for Sn rich solutions—indirect evidence of higher Sn incorporation compared to Zn. (**b**) shows the interdependence of the film composition on solution cation ratio and growth temperature. The lower the temperature the larger the discrepancy between the nominal solution cation ratio and the measured cation ratio in the thin film.

**a-ZnSnO_3_:** For a third ternary material investigated here, a-ZnSnO_3_, we observe an intermediate case. Here, the presence of the Sn (and Cl) ions in the gas phase, or at the surface, inhibits Zn incorporation, but does not fully suppress it, while the presence of the Zn ions promotes Sn incorporation. There is an increase in the overall growth rate compared to expectations from the study of the binary oxides SnO_2_ and ZnO using chloride precursors. Figure 7 illustrates this by showing the influence of the Zn:Sn ratio on total film thickness and the interdependency of solution Zn:Sn ratio and temperature on the film stoichiometry.

The example shown here is from a more extensive study where growth time, growth temperature, and solution cation ratio were systematically varied within a Box–Behnken design of experiment allowing for the shown parabolic extrapolation of the interdependence of the film stoichiometry and other properties on growth conditions summarised in the SI. It is worth highlighting that the observed behaviour when growing the ternary material with both cations being present in solution and gas phase is dramatically different from the expectations based on the assessment of the growth rates of the binary oxides. Figure 3 would suggest that lowering the temperature would increase Zn incorporation as the highest growth rates were found around 320 °C, yet, we find that at lower temperatures films tend to be even more Sn-rich. Unfortunately, the method employed for the single precursors is limited to temperatures below 350 °C, while ideal growth conditions for a-ZTO are around 380 °C, making a more direct comparison not feasible.

For the specific example of a-ZTO grown from chlorides, as a result, we observe a non-stoichiometric transfer of the solution cation ratio (y-axis in Figure 7b) to the thin-film composition measured by XPS (colour scale in Figure 7b). This is most dominant at lower temperatures as for instance a 50:50 nominal Sn:Zn ratio leads to a 75:25 ratio at 350 °C while at 410 °C already a 70:30 ratio is found in the film. Increasing the temperature further is expected to result in films even closer to stoichiometric transfer. Indeed, in previous work synthesising crystalline cubic spinel Zn_2_SnO_4_ using chloride precursors at much higher growth temperatures, stoichiometric transfer is observed. The latter was confirmed by the presence of a phase pure XRD pattern [10]. However, in that case, SnCl_4_ was used, not the SnCl_2_ employed here. In our system, we found SnCl_2_ to be easier to handle and leading to higher reaction yields, hence the choice of that precursor may contribute to the observed increased Sn incorporation.

In summary—once a ternary material is grown—surface reactions can significantly alter growth and incorporation rates. As a result, known growth rates of constituent binary materials may not be enough to predict the behaviour of ternary systems.

## 4. Influence of Spray Geometry

**Figure 8:**
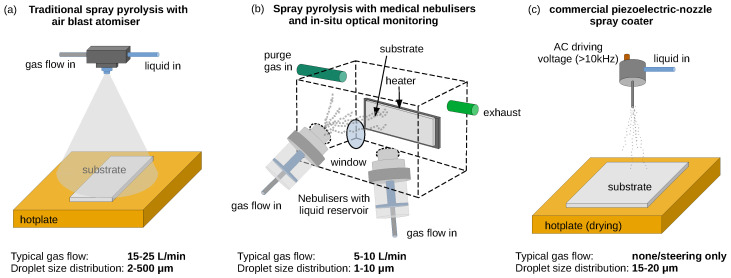
Schematic illustration of different system geometries used for spray pyrolysis. (**a**) illustrates the basic setup with an air-blast nozzle and hot-plate. This type of nozzle requires substantial gas flow and has a broad droplet size distribution. (**b**) illustrates our modified setup requiring much lower gas flows and a narrower droplet size distribution. The small droplet sizes lead to an air-suspended mist. This configuration is also referred to as Mist-CVD. Our system allows spraying from two separate nebulisers to allow either alternating sequences or simultaneous spraying of different precursor solutions. (**c**) the narrowest droplet size distribution is achieved by piezoelectrically driven nebulisers, which require no gas flow to create the droplets. Those are often found in commercial spray coaters but in combination with hot-plates can also be used for spray pyrolysis. (**a**,**c**) can be used in open geometries, while (**b**) requires a chamber to keep the precursor mist within the sample area.

Spray pyrolysis is easily set up and the most basic systems consist of an air-blast nozzle spraying onto a hot-plate (see Figure 8a), while more sophisticated systems use ultrasonic nozzles, nebulisers, mass flow controllers for the gas supply, and PID-controlled specialised low-heat-capacity heaters. While the set-up of spray pyrolysis is simple, the actual physical processes determining the droplet size in the nebuliser, the rate of solvent evaporation, the temperature gradient, and precursor concentration gradients within the spray system are far from simple. Perednis and Gauckler [3] have outlined the different processes leading to various growth modes from liquid–solid reactions, surface chemistry-driven CVD, to dry-particle formation in the gas phase. This complexity, caused by the large droplet size distribution in many simpler systems, often leads to difficulties in reproducing growth recipes from the literature. Typically these are given in terms of substrate temperature, solvent type, precursor molarity, spray rate, and time. Depending on the exact dimensions, nozzle type, and heater control, the resulting film properties can therefore vary substantially.

Growth temperatures are typically given by PID-controlled heater set-points of either hot-plates or dedicated ceramic heaters. The available power and heat capacity of the heater, as well as the gas flow from the spray nozzle and thermal conductance of the substrate will define the actual surface temperature the spray pyrolysis process occurs in. The key temperature for the synthesis process is the substrate surface temperature under spray conditions. In spray pyrolysis, however, measurements of the surface temperature are very difficult as pyrometry does not give reliable results due to the spray mist. Controlling the heater temperature is therefore the most common technical control parameter. When comparing sample properties as function of temperature within the same system, the systematic difference between heater and substrate surface is less important. When comparing growth recipes between different systems they can become more important.

**Figure 9:**
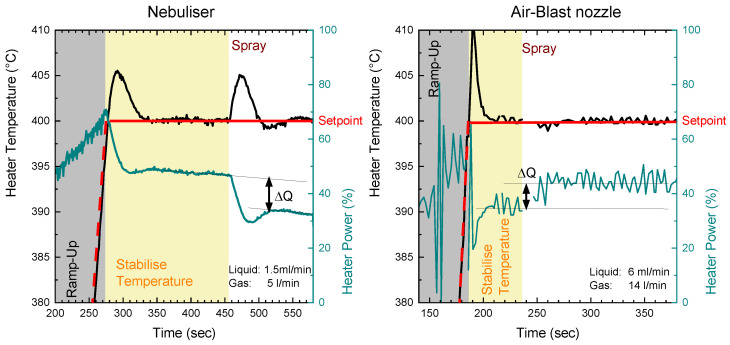
Temperature and heater power readings at the start of a spray process taken in two different spray set-ups using either an ultrasonic nebuliser or a traditional air-blast nozzle. The chamber geometries, liquid as well as gas flow differ significantly. Both runs use methanol as the solvent and 400 °C as the set-point temperature. It can be seen that for the more gentle nebuliser with less air flow, there is an effective heat transfer from the burning of the solvent to the sample surface and subsequently the heater itself. For the air-blast nozzle, this effect is compensated by the much higher air flow when the spraying is activated.

Figure 9 shows the recorded heater temperature and heater power at the beginning of a growth cycle using two different spray systems. In both cases, the solvent was methanol, the heater itself a ceramic heater with low heat capacity. If larger hot-plates are used within the spray pyrolysis system, those nozzle-type-induced surface temperature differences will be even larger, as the heat capacity of larger hot-plates slows down the PID control of the temperature. In this example, methanol was used as a solvent which partially compensates the surface cooling effect of the gas-flow as the solvent burning process transfers heat to the sample surface. For the nebuliser, using significantly lower gas flows, this is even seen as a brief increase in heater temperature until the PID control reduces the required power to the heater to maintain the set-point temperature. For the air-blast nozzle, the heater power had to be increased to maintain a stable heater temperature, caused by the much higher gas flow required to spray the solution. For other solvents, this effect will differ; ethanol provides less heating power, while water provides none at all, leading to cooler conditions above the sample [61].

As shown, the temperature in the reaction zone is the key parameter for the precursor decomposition, which, in itself is the key parameter when balancing the surface chemistry for ternary materials, as at least two different cation precursors are needed. It is therefore also important to evaluate any spatial differences in the surface temperature within a spray pyrolysis system. As an example, we looked into the growth of a simple test binary oxide Cu_2_O within the small nebuliser-based spray system. With the nebuliser, we have established that growth conditions are CVD-like and that the cooling from the gas flow from the nebuliser can be compensated by the burning of the solvent. With the binary compound, any observed thickness variation within a sample is hence reflective of surface temperature variations across the sample surface.

Since our existing XPS only integrates over a large area and lacks mapping capabilities, it was not possible to use it to illustrate the effect of spray geometry on the stoichiometry. We therefore employed UV-VIS transmission measurements where we can map with mm spatial resolution to illustrate the impact of nozzle choice. Film thicknesses were extracted from each UV-VIS spectrum by a neural network trained on synthetic data (details of the method provided in Supplementary Materials, Section S2.3).

**Figure 10:**
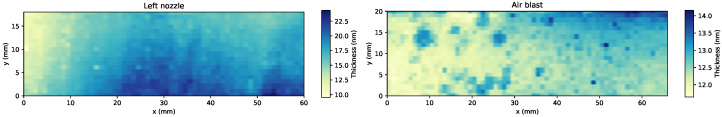
Thickness maps with 1 mm spatial resolution of two Cu_2_O films grown using a nebuliser-based and an air-blast system (see Figure 8). The overall thickness varies slightly but relative variations can be compared. For the nebuliser system, there are gradual variations (lower edge thicker, top left corner thinnest), while the air-blast system with the larger spray radius and much bigger heater is globally more homogeneous but has many local variations due to the impact of larger, not fully evaporated droplets.

Figure 10 shows the thickness variations with two Cu_2_O films grown in different chamber geometries. One used a nebuliser spraying from the left-hand-side of the chamber, the other an air-blast system spraying onto a larger hot-plate. In this nebuliser system, there are variations across the sample surface—these are not caused by the directed gas flow but the overall gas flow (evacuated exhaust on the right), convection (top hotter than bottom), and heat conduction between heater and glass slide (clamped on the left, best heat transfer). As a result, there are thickness variations across the sample, which are reproducible and consistent across samples. In contrast, the air-blast nozzle with the large heater shows globally better homogeneity but large local thickness variation, which are caused by the occasional impact of large droplets depositing much more precursor material in small areas. Those variations are of a stochastic nature and happen at different positions for each growth run.

**Figure 11:**
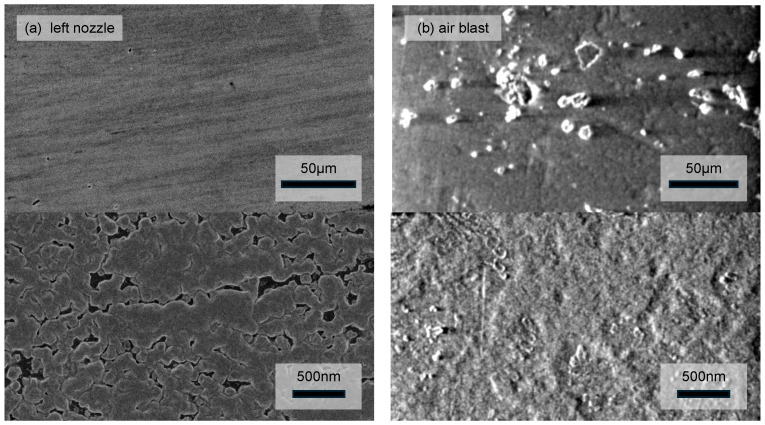
Scanning Electron Microscopy images of the (**a**) nebuliser-based system and (**b**) air-blast system on a micro and nano scale. The air-blast system shows more local variations of particle sizes, with some unevaporated droplets still on the micro scale, where the nebuliser-based system looks more homogenous. At higher magnifications, the nanostructure of the nebuliser-based system begins to reveal much more structure in comparison to the air-blast geometry. This highlights that there are systematic differences in the resulting film across SP geometries, and it is not a case of one superior method of fabrication, but rather differences in each method.

Figure 11 shows the Scanning Electron Microscope (SEM) images of the left nozzle and air-blast samples (details of the method provided in Supplementary Materials, Section S2.4). In agreement with the 1 mm spatially resolved transmission maps, the air-blast system shows more global homogeneity and, once again, is impacted by larger droplets of material. On the nanoscale, the nebuliser-based system provides more nanostructure, and hence a greater lack of homogeneity in comparison to the air-blast system, which may be the result of a smaller droplet size, shorter distance from nebuliser to the substrate, or even film cracking due to a faster sample cool-down with the small low-heat-capacity heaters. These differences as a function of magnification showcase some of the structural differences of the resulting film in different SP geometries. While the exact details will be different for each material grown, a difference in film quality when using various SP geometries should be expected. This becomes crucial when reproducing growth recipes from the literature, as films can vary significantly.

The desorption of Cu-suboxides from the surface (see Figure 1) leads to a case where a cooler substrate surface leads to a thicker Cu_2_O film. The observed thickness variations in Figure 10 for the nebuliser system are therefore indirectly proportional to the real surface temperature. For the air-blast-grown sample, the underlying global variations are also temperature-related, while the small scale “droplet” shaped variations are caused by the larger droplet size distribution.

For binary oxides such as Cu_2_O, both temperature- and droplet-induced variations only lead to thickness inhomogeneities. In ternary systems, with different precursors reacting differently and potentially different temperature dependence of incorporation, this mechanism is therefore also expected to lead to spatial stoichiometry variations.

## 5. Conclusions

We have shown in this work how the complex growth process of spray pyrolysis affects the synthesis of selected ternary oxides. We investigated individual precursors’ decomposition temperatures employing refractive index sensing on Au-dimer decorated substrates to understand the origin of non-stoichiometric precursor transfer from solution to the film. We provided experimental examples of surface reactions enabling or suppressing the film formation for three ternary oxide systems, CuCrO_2_, SnTiO_*x*_, and a-ZnSnO_3_, to highlight the behaviour and dynamics of multi-precursor film growth. We demonstrated thickness variations for Cu_2_O films depending on the type of spray geometry used, while spray pyrolysis remains an attractive method due to the ease of solution preparation and overall simplicity of the set-up, we have outlined all the challenges of the method when working with ternary or even more complex oxides. Differences in solubility, solution-cross-reaction, precursor decomposition, on-surface reactions, suboxide desorption, and geometry- or nebuliser-choice-induced temperature gradients can all lead to variations in the stoichiometry of the grown film compared to the nominal solution concentrations. These effects are especially pronounced at lower deposition temperatures.

Once all these effects are considered and growth conditions of different systems carefully adjusted to translate nominal growth temperature to the actual sample surface temperature, spray pyrolysis can be employed reliably. The in-depth understanding of these processes can now also be deliberately employed in the future to synthesise samples with controlled composition gradients for, i.e., material screening projects. By enforcing temperature gradients or preventing gas mixing within a nebuliser-based system, the synthesis of composition-spread samples by spray pyrolysis becomes feasible.

## Figures and Tables

**Table 1:** Overview of some spray pyrolysis precursors, their solubility in H_2_O (highest molarity, S_*W*_), ethanol (S_*Eth*_), and methanol (S_*Meth*_, qualitative only: s = soluble, sl = slightly soluble, i = insoluble). The melting point of the salt and, if available, the threshold for thermal decomposition is listed. The selection is based on salts with relatively low decomposition temperatures and found soluble in either water, ethanol, or methanol. This makes them ideal candidates for spray pyrolysis precursors.

Name	Formula	S_*W*_ (M)	S_*Eth*_	S_*Meth*_	mp (°C)	T_*d*_ (°C)	Ref.
**Al**							
Aluminium acetylacetonate	Al(C_5_H_7_O_2_)_3_	i	sl	sl	190	200	[24]
Aluminium chloride hexahydrate	AlCl_3_·6H_2_O	1.9	s	s	100	90	[25,26]
Aluminium nitrate nonahydrate	Al(NO_3_)_3_·9H_2_O	2.2	s	s	73	135	[25,27]
**Ba**							
Barium hydroxide octahydrate	Ba(OH)_2_·8H_2_O	0.16	sl	s	78	-	[25]
**Bi**							
Bismuth trichloride	BiCl_3_	-	s	-	234	-	[25]
**Ca**							
Calcium nitrite	Ca(NO_2_)_2_	7.2	sl	-	392	-	[25]
**Cu**							
Copper(II) acetylacetonate	Cu(O_2_C_5_H_7_)_2_	0.01	sl	sl	286	200	[25,28,29]
Copper(II) trifluoroacetylacetonate	Cu(C_5_H_4_F_3_O_2_)_2_	-	s	s	197	250–300	[25,30]
Copper(II) chloride dihydrate	CuCl_2_·2H_2_O	4.5	s	s	100	-	[25]
Copper(II) D-gluconate	C1_2_H_22_CuO_14_	0.07	sl	sl	156	170	[25,31]
Copper(II) nitrate trihydrate	Cu(NO_3_)_2_·3H_2_O	6.0	s	s	114	135	[25,32]
Copper(II) perchlorate hexahydrate	Cu(ClO_4_)_2_·6H_2_O	3.9	s	s	82	120	[25,33]
Copper(II) sulfate pentahydrate	CuSO_4_·5H_2_O	0.88	sl	s	110	63	[25,34]
**Cr**							
Chromium(III) acetylacetonate	Cr(C_5_H_7_O_2_)_3_	i	sl	sl	216	325	[25,28]
Chromium(III) chloride hexahydrate	CrCl_3_·6H_2_O	2.2	s	s	83	85	[25,35]
Chromium(III) nitrate nonahydrate	Cr(NO_3_)_3_·9H_2_O	2.0	s	s	66	110	[25,36]
Chromium(II) sulfate pentahydrate	CrSO_4_·5H_2_O	0.9	sl	s	100	-	[25]
**Fe**							
Iron(III) acetylacetonate	Fe(C_5_H_7_O_2_)_3_	0.01	s	s	188	180	[25,28,37]
Iron(III) chloride	FeCl_3_	5.6	s	s	304	316	[25,38]
Iron(II) sulfate monohydrate	FeSO_4_·H_2_O	1.7	i	-	300	225	[25,39]
**K**							
Potassium acetate	KC_2_H_3_O_2_	27	s	-	309	-	[25]
Potassium dihydrogen phosphate	KH_2_PO_4_	1.8	s	-	-	253	[25]
Potassium pyrosulfate	K_2_S_2_O_7_	s	-	-	325	-	[25]
**La**							
Lanthanum nitrate hexahydrate	La(NO_3_)_3_·6H_2_O	4.6	s	s	70	200,380	[25,40,41]
Lanthanum perchlorate hexahydrate	La(ClO_4_)_3_·6H_2_O	4.4	s	s	100	200	[25,42]
**Li**							
Lithium acetate	LiC_2_H_3_O_2_	6.8	s	s	286	400	[25,43]
Lithium formate monohydrate	Li(CHO_2_)·H_2_O	4.8	-	-	94	-	[44]
Lithium iodide trihydrate	LiI·3H_2_O	8.8	s	s	73	200	[25,45]
Lithium nitrate	LiNO_3_	-	s	-	253	-	[25]
Lithium perchlorate trihydrate	LiClO_4_·3H_2_O	4.0	s	s	95	400	[25,46]
**Mg**							
Magnesium chloride hexahydrate	MgCl_2_·6H_2_O	3.1	s	s	58	100	[25,47]
**Mn**							
Manganese(II) nitrate hexahydrate	Mn(NO_3_)_2_·6H_2_O	5.6	s	s	32	180	[25,48]
Manganese(II) chloride tetrahydrate	MnCl_2_·4H_2_O	10	s	s	58	230	[25,49]
**Rb**							
Rubidium hydrogen sulfate	RbSO_4_	s	-	-	208		[25]
Rubidium nitrate	RbNO_3_	4.4	s	-	310	-	[25]
**Sn**							
Tin(II) 2-ethylhexanoate	Sn(C_8_H_15_O_2_)_2_	-	s	s	<0	200	[25,50]
Tin(II) chloride	SnCl_2_	4.7	s	s	246	>420	[25,51]
**Sr**							
Strontium chloride hexahydrate	SrCl_2_·6H_2_O	-	s	-	100	100	[25]
Strontium nitrite	Sr(NO)_2_	3.4	-	-	-	240	[25]
Strontium iodide hexahydrate	SrI_2_·6H_2_O	-	s	-	120	120	[25]
**Ta**							
Tantalum ethoxide	Ta(OC_2_H_5_)_5_	-	s	s	21	250	[25,52]
Tantalum(V) chloride	TaCl_5_	s	s	-	216.6	-	[25]
**Ti**							
Titanium (IV) oxysulfate solution	Ti_2_(SO_4_)_3_	-	s	-	-	-	[25]
Titanium chloride	TiCl_3_	s	s	s	-	425	[25,53]
Titanium methoxide	Ti(OCH_3_)_4_	-	sl	s	205	300	[25]
**V**							
Vanadyl acetylacetonate	VO(C_5_H_7_O_2_)_2_	-	s	s	235	290	[54]
**Zn**							
Zinc acetate dihydrate	Zn(C_2_H_3_O_2_)_2_·(H_2_O)_2_	1.4	s	s	237	250	[25,55,56]
Zinc acetylacetonate hydrate	Zn(C_5_H_7_O_2_)_2_·xH_2_O	0.025	s	s	130	130	[25,57]
Zinc chloride	ZnCl_2_	-	-	-	290	450	[25,58]
Zinc nitrate hexahydrate	Zn(NO_3_)_2_·6H_2_O	-	-	-	36	-	[25]
**Zr**							
Zirconium(IV) chloride	ZrCl_4_	s	s	s	437	-	[25]
Zirconyl chloride octahydrate	ZrOCl_2_·8H_2_O	322.252	s	-	400	400	[25]

## Data Availability

The original contributions presented in the study are included in the article/Supplementary Materials, further inquiries can be directed to the corresponding authors.

## Supplementary Materials

The following supporting information can be downloaded at: https://www.mdpi.com/article/10.3390/ma19153305/s1, Figure S1: XPS spectrum and XRD pattern for a SnTiO_x_ sample; Figure S2: XPS spectra in the Cl 2p region for various SnTiO_x_ samples. Refs [68,69,70,71,72] are included in the Supplementary Materials.
