# Supplementary material for: Spray Pyrolysis of Ternary Oxides: From Precursor Selection to Surface Reactions
Source: Materials (Basel). 2026 Aug 4;19(15):3305. doi: 10.3390/ma19153305 (PMC13468541; doi:10.3390/ma19153305)
Supplement: Supplementary file 1 [file materials-19-03305-s001.zip › materials-4393656-supplementary.pdf]

Supplementary Material

Spray pyrolysis of ternary oxides: from precursor selection to surface reactions

Karsten Fleischer[1,2,*], Priyanka Bhatnagar[1], Ciarán Cooling[1], Eva Gurley[1], Dominik Jakobczak[1], Ainur Zhussupbekova[1,2,3]

[1] School of Physical Sciences, Dublin City University, Dublin D09 V209, Ireland
[2] School of Physics and Centre for Research on Adaptive Nanostructures and Nanodevices (CRANN), Trinity College Dublin, Dublin D02 PN40, Ireland
[3] L.N. Gumilyov Eurasian National University, Astana 010000, Kazakhstan.
* Correspondence: karsten.fleischer@dcu.ie; ainur.zhussupbekova@dcu.ie

S1. Thin film deposition

Deposition of thin films discussed in the manuscript have been performed in the mist-Chemical vapour deposition (CVD) spray pyrolysis chamber previously described in other work [1]. Selected comparisons have been made with films grown in an air-blast system described in [2]. The design of the "mist" system with an optical window provided an access to perform the refractive index sensing using reflection anisotropy spectroscopy (RAS) to reveal individual Cu, Cr, Zn, and Sn precursors decomposition temperatures depicted in Figures 1-4 of the manuscript. Details of the methodology for sensing with Au dimer decorated substrates previously reported [1].

In order to avoid cross contamination between the different materials the studies on each system have been performed in separate chambers. Chambers have been cleaned between runs and for all studies a “conditioning run” with pure solvents after chamber cleaning is done.

$SnTiO_x$ samples discussed in Figure 6 of the manuscript were synthesized using both organic and inorganic precursors (Sigma Aldrich) dissolved in ethanol. The inorganic system includes Titanium (III) chloride and Tin (II) chloride, while Titanium (IV) methoxide and Tin (II) ethylhexanoate serve as the organic precursors. All samples were prepared on glass substrates with fixed molarity of 0.1M, growth temperature of 380℃, carrier flow of 5% and deposition time of 600 s.

a-ZTO thin films discussed in Figure 7 of the manuscript were deposited using Tin (II) chloride and Zn (II) chloride precursor dissolved in ethanol with various molarities of 0.07, 0.1 & 0.13M. Samples were deposited on glass substrates, with the solutions mixed in the nebuliser in various ratios between 30-70% Tin to Zinc, heater temperatures ranging from 350 - 410°C with compressed air as the carrier gas.

The Cu_2O samples discussed in Figure 10 included for the comparison of thickness homogeneity across different spray systems were deposited using copper acetylacetonates

in two different spray geometries. Different precursors, solution molarities, and spray times were used across the two geometries so that film thickness, and hence, homogeneity was comparable. This highlights the difficulties in reproducing growth recipes from system to system, and further explains that care should be taken when employing spray pyrolysis recipes taken from the literature. While spray time and molarity have a major effect on the overall thickness of the film, temperature and carrier gas composition tend to induce phase related changes in the samples [3]. Therefore, effort was made to ensure these parameters were consistent across geometries.

The 'left nozzle' sample used 0.05M of Copper(II) Trifluoroacetylacetonate with 97% purity from Sigma Aldrich (Merck) dissolved in methanol. The spray used a carrier flow of 4 L/min consisting of a mixture of N_2 and compressed air, calibrated to supply 5% O_2 flowing through the nebuliser. The sample was deposited on a standard glass slide for 240s at 300℃, and was brought back to room temperature slowly in a full nitrogen environment to avoid overoxidation of the cupric oxide.

The 'air blast' sample was deposited in a custom built confined environment (Abbess 18″ Cube), employing an air blast atomizer nozzle placed at a distance of 29 cm from the deposition substrate (further details of the system available in other work [2]) using 0.01M of Copper(II) Acetylacetonate with 97% purity from Sigma Aldrich (Merck) dissolved in methanol. The spray used a carrier flow of 25 L/min consisting of a mixture of N_2 and compressed air, calibrated to supply 5% O_2 flowing into the chamber. Deposition was performed on a standard glass slide at 300℃ for 600s.

S2. Sample characterisation

S2.1 X-ray diffraction (XRD)

Crystalline structure and phase evolution of the deposited films were investigated employing a Bruker D8 Advance XRD system with a Cu Kα X-ray source. Due to the low temperatures employed in the synthesis, all samples except Cu_xCrO_2 and Cu_2O [3, 4] are of amorphous nature with no discernible diffraction peaks apart from those originating from the glass substrate and PMMA sample holder, indicating that the as-deposited films are X-ray amorphous. A representative scan for $SnTiO_x$ is shown as an example in Figure S1(b).

S2.2 Compositional analysis via X-ray photoelectron spectroscopy (XPS)

With the lack of crystalline order in most films, XRD and XRD-peak positions can not be employed to analyse lattice constants and therefore indirectly stoichiometry. XPS was therefore employed for all systems to determine the elemental composition, chemical states and surface stoichiometry of the samples. The XPS uses a monochromatic Al Kα (hv = 1486.7 eV) X-ray source with a 128 channel Argus CU detector. The XPS system was connected to an plasma assisted Atomic layer deposition (PA-ALD) chamber where the samples were treated in-situ by low energy oxygen plasmas for 2 minutes to remove surface

contamination from the atmospheric transfer prior to the measurement. The XPS elemental ratios were determined by analysing integrated peak counts, transfer function, elemental sensitivities and the expected energy dependent penetration depth [5]. The validity of the procedure was verified by analysing highly crystalline bulk $SrTiO_3$ (001) single crystal substrates from MTI Corporation. We have to note that our XPS can not be used to assess the Oxygen content reliably as the necessary air exposure leads to surface oxidation (or hydroxide formation). While the oxygen plasma treatment removes some of the carbon and hydroxide contamination it can also affect the oxygen content. We therefore only discuss the cation ratios.

The $SnTiO_x$ sample prepared using Sn(II) 2-ethylhexanoate and Ti methoxide (IV) precursor solution (chloride-free) is shown in Figure S1. The XPS spectrum (Figure S1(a)) confirms the successful incorporation of Ti, Sn, and O in the deposited film, with an approximate Sn:Ti atomic ratio of 1:1, demonstrating the formation of the desired material stoichiometry. The corresponding XRD pattern of the same sample (Figure S1(b) does not exhibit any discernible diffraction peaks though. The broad structure and single peak seen originate from the glass substrate and PMMA sample holder. This indicates that the as-deposited film $SnTiO_x$ is X-ray amorphous. Therefore, XPS provides the primary evidence for the composition of the $SnTiO_x$ films.

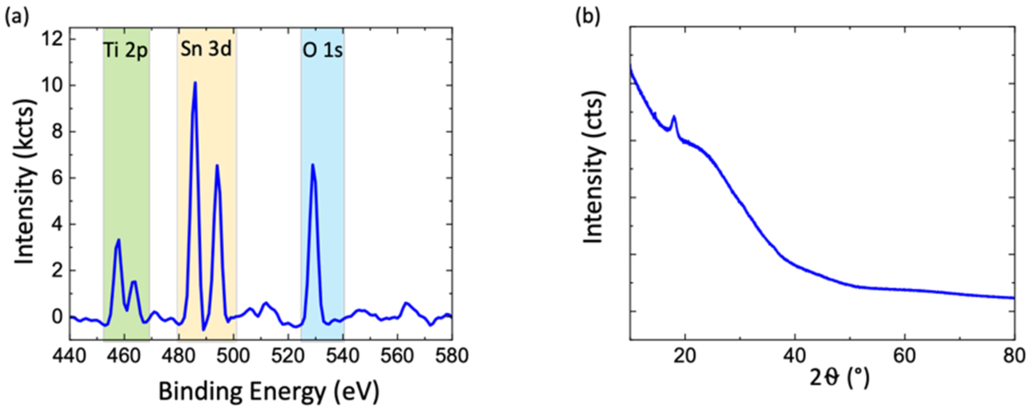

***Figure S1**. (a) XPS spectrum for $SnTiO_x$ sample (grown using Ti methoxide (IV) and Sn(II) 2-ethylhexanoate precursor solution), showing the presence of Ti, Sn and O elements in the fabricated thin film with Sn:Ti atomic ratio of roughly 1:1 (b) XRD of the same $SnTiO_x$ sample illustrating the highly amorphous nature of the film.*

The XPS spectra of films deposited using $SnCl_2/TiCl_3$ precursor solutions (Figure S2(a)) showed Cl 2p signals**.** These spectra were acquired before surface cleaning to determine if part of the detected chlorine may originate from unreacted precursor species adsorbed on the film surface. In contrast, no detectable Cl signal was observed for films deposited using the $SnCl_2$/Ti methoxide precursor combination, indicating the presence of Cl only with $TiCl_3$ precursor. To further investigate the origin of the Cl signal, O_2 plasma cleaning

was performed on a film deposited using only the $TiCl_3$ precursor. Following plasma treatment, the Cl intensity decreased as shown in Figure S2(b), demonstrating that a fraction of the detected chlorine was associated with the film surface. However, a weak Cl signal remained after cleaning. Further measurements would be needed to determine if this is due to incorporation or incomplete surface cleaning (i.e. due to shadowing effects in rougher samples). Interestingly, no Ti signal was detected in either the $TiCl_3$ only film or the $SnCl_2$/ $TiCl_3$ film, whereas Ti was detected when chloride-free precursors (Ti methoxide and Sn(II) 2-ethylhexanoate) were employed. These observations support our assumption that chloride-containing precursor systems strongly influence Ti incorporation during spray pyrolysis, although the exact mechanism remains to be established. In summary chloride incorporation is only suspected in samples grown with $TiCl_3$. All other samples, including all a-ZTO samples grown with $SnCl_2$ and ZnCl did not show any measurable Cl XPS signal. With detection limits in the order of 0.1% we can however not exclude incorporation on a lower level.

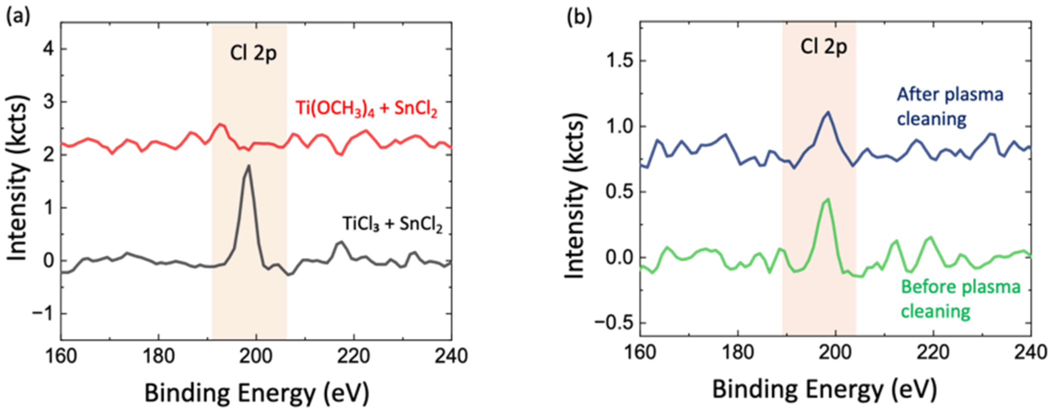

Figure S2. *(a) XPS spectra in the Cl 2p region for films deposited using $SnCl_2$/ $TiCl_3$ and $SnCl_2$/ Ti (IV) methoxide precursor systems (b) XPS spectra of the $TiCl_3$-derived film before and after O_2 plasma cleaning.*

The XPS analysis of a-ZTO samples shown in the Figure 7 of the main article are part of a more comprehensive study on optimising spray pyrolysis grown a-ZTO growth by employing a design of experiment (DoE) concept to systematically screen three independent growth parameters (temperature T, total molarity M, Sn/Zn solution ratio R) [6]. The concept involves a Box-Behnken DoE where the three parameters are varied in a "higher/lower" step from a central point. Here T=380, M=0.1, and R=50 was chosen as the central point with steps of ΔT=±30, ΔM=±0.03, ΔR=±20. All parameters are varied systematically creating a set of 16 different runs, the run order is randomised to avoid systematic errors, and the central point is measured multiple times to assess reproducibility. For more details on the Box-Behnken design method itself we would like to refer to a dedicated review and example of its use for SP [7,8]. After the full set of samples is grown various film properties are measured for all samples such as thickness, sheet resistance, UV-VIS transmission, carrier concentration,

carrier mobility, and most relevant for this work the Sn/Zn ratio (by XPS) within the samples. The influence of the three parameters varied on the individual film properties is then modelled via quadratic expressions and least square fits of these models to the entire sample set. This allows visualization of the dependence of a given property as a function of two parameters in the form of contour plots, as well as predictions of ideal growth parameters to achieve a certain film property. Figure 7 in the main article is such a visualisation of the Sn/Zn ratio found in the film by XPS as a function of growth temperature and Sn/Zn ratio in solution to illustrate the non-stoichiometric transfer in the case of a-ZTO for the specific chloride precursors.

S2.3 Spatially resolved UV-VIS spectrophotometry using machine learning models.

To illustrate possible temperature gradients across the samples grown in different spray pyrolysis systems (air blast and nebuliser) we required an indirect assessment as surface temperatures are inaccessible in spray pyrolysis, particularly spatially resolved. We therefore use the growth of a simple binary oxide Cu_2O as an indicator because our study (see Figure 1, main article) revealed a temperature dependent growth rate around 300℃. Temperature variations across the sample surface will therefore materialise as thickness variations across the sample. Cu_2O was chosen, as the material has a bandgap of 2.1eV hence creating absorption features in the UV-VIS range easily probed by UV-VIS spectrophotometry. The thickness maps shown in Figure 10 of the main text were measured with a inhouse-built UV-Vis transmission mapping spectrophotometer (1mm spatial resolution, tungsten broad band lamp, fibre coupled StellarNet EPP2000 spectrometer, Eksma Optics motorised translation stages). This technique was used to generate the spatially resolved transmission measurements of Cu_2O samples grown in different deposition chambers using different geometries as discussed in Figure 8 of the main text. Samples were mounted on a motorised stage and stepped in a raster pattern at 1 mm resolution, with a transmission spectrum recorded over 400-850 nm at each position. Spectra containing unphysical transmission values or affected by the sample mount and substrate edges were removed by an automated filter before analysis. Film thickness was extracted from each accepted spectrum by a neural network trained on synthetic data. Transmission spectra of the air/Cu_2O/glass system were simulated using the transfer matrix method at 10,001 thickness values between 0 and 100 nm, with the glass substrate treated as an incoherent layer and the Cu_2O dielectric function described by a Lorentz oscillator model fitted to published spectroscopic ellipsometry data for bulk crystalline Cu_2O. An ensemble of ten independently initialised feedforward neural networks with residual connections was trained on the simulated spectra, and the median of the ten predictions was taken as the film thickness at each map position. On a held-out synthetic test set the ensemble achieved a root mean square error of 0.37 nm. Applied to a reference Cu_2O film, the mean predicted thickness of 10.3 ± 2.3 nm was consistent with an independent spectroscopic ellipsometry measurement of 11.2 ± 1.2 nm at the substrate centre, and the predicted maps reproduced the direction of the thickness gradient observed in a lower spatial resolution (5mm) ellipsometry map of the same film.

S2.4 Field-Emission Scanning Electron Microscopy (FESEM)

FESEM images of two Cu_2O films grown in different geometries were taken to show differences in homogeneity at the micro and nano scale. Samples were mounted on the standard STEM holder, and stuck down with a small carbon pad. A drop of colloidal graphite was added to the very edge of the sample to avoid sample charging, given the insulating nature of the glass substrate. The images were acquired using The Hitachi S-5500 FESEM using a low energy 1KV electron beam and 10μA of current. The beam was tuned and focused on the sample, allowing it to stabilise for about an hour before images were acquired.

References.

1. Zhussupbekova, A.; Zhussupbekov, K.; Verre, R.; Caffrey, D.; Shiel, K.; Shvets, I.V.; Fleischer, K. An in Situ Study of Precursor Decomposition via Refractive Index Sensing in P-Type Transparent Copper Chromium Oxide. Chemistry of Materials 2022, 34, 3020–3027. https://doi.org/10.1021/acs.chemmater.1c03910.
2. Arca e.; K. Fleischer; Shevts I.V. Influence of the Precursors and Chemical Composition of the Solution on the Properties of ZnO Thin Films Grown by Spray Pyrolysis, Journal of Physical Chemistry C 2009, 113, 50. https://doi.org/10.1021/jp907990z
3. Gurley, E.; Fleischer, K. Controlled Chemical Phase Engineering in Spray Pyrolysis of Thin Cu, Cu2O, and CuO Films. in preparation 2026.
4. Farrell L., Norton E., Smith C., Caffrey D., Shvets I.V., Fleischer K. Synthesis of nanocrystalline Cu deficient CuCrO2–a high figure of merit p-type transparent semiconductor. Journal of Materials Chemistry C 2015, 126, 4. https://doi.org/10.1039/c5tc03161c
5. Omicron Multiprobe Manual, CasaXPS user manual.
6. Cooling, C., Gurley, E. & Fleischer, K. An optimisation procedure for spray pyrolysis grown amorphous zinc tin oxide using a Box-Behnken design of experiment. *in preparation* (2026).
7. Ferreira, S. L. C. et al. Box-Behnken design: An alternative for the optimization of analytical methods. Analytica Chimica Acta 597, 179–186 (2007). https://doi.org/10.1016/j.aca.2007.07.011
8. Mariño-Gámez, A. E. et al. Design of Experiments (DoE) Approach for Optimizing the Processing and Manufacturing Parameters of SnO_2 Thin Films via Ultrasonic Pyrolytic Deposition. J. Compos. Sci. 9, 555 (2025). https://doi.org/10.3390/jcs9100555
